# Strengths, Pitfalls, and Lessons from Longitudinal Childhood Asthma Cohorts of Children Followed Up into Adult Life

**DOI:** 10.1155/2016/2694060

**Published:** 2016-10-31

**Authors:** Andrew Tai

**Affiliations:** ^1^Respiratory and Sleep Medicine, Women's and Children's Hospital, King William Road, North Adelaide, SA 5006, Australia; ^2^Robinson Research Institute, University of Adelaide, SA 5005, Australia

## Abstract

Asthma is a common problem worldwide and longitudinal studies of children followed up into adult life enable the assessment of clinical outcomes, examine the pattern of lung function outcomes, and importantly provide insight into aetiology and prognosis for patients with asthma. The aim of this review is to examine the major childhood asthma cohort studies which have continued into adult life, describing the strengths and weaknesses and the lessons that can be learnt regarding pathophysiology and potential future directions for research.

## 1. Introduction

Asthma is one of the commonest conditions affecting the worldwide community. Monitoring the long-term patterns of childhood asthma into adult life has provided important information on clinical outcomes, highlighted lung function trends over time, enabled investigations into the aetiology and pathophysiology of asthma, measured treatment responses, and, importantly, provided prognostic information for families and their children. In the last 2 decades, there have been few prospective community studies which have explored the long-term outcome of childhood asthma into adult life. Some of the studies have followed up children to adolescence [1] and young adulthood [2] with limited reports describing patterns into adult life [3, 4].

The lung function outcome in adult life is determined by 2 factors. Firstly lung growth occurs in children to young adult life and suboptimal growth may be potentially impacted by childhood diseases such as asthma and premature gestation. Secondly, following maximal lung growth, there is a decline that occurs in adult life and this decline may be accelerated by insults such as smoking.

The purpose of this review is to highlight the strengths, weaknesses, and lessons which can be learnt from the landmark studies of children with asthma who have been followed up into adult life.* This review will focus on five specific longitudinal cohorts which have followed up children with asthma prospectively from childhood to varying ages of adult life (Table 1). These specific cohorts have longitudinal clinical data and lung function measurements which enables a thorough comparison in order to establish key outcomes, learning points, and future research concepts. There are many additional birth cohorts which have not reached adult life and will therefore not be discussed for the purposes of this review*. This review will highlight clinical factors that determine the persistence of asthma from childhood into adult life and examine the lung function trends from childhood to adult life and the potential impact of disease such as asthma and the interaction with smoking.

## 2. Melbourne Asthma Cohort

In 1964, the late Williams initiated a community-based, prospective study of a group of children who were born in Melbourne from the 1957 birth cohort with a history of wheezing [5]. The original aim of the study was to determine the prevalence and describe the natural history of asthma and wheezy bronchitis in children. The term “wheezy bronchitis” was used in the 1960s for episodes of wheezing associated with symptoms of an underlying respiratory tract infection. William's hypothesis was that “wheezy bronchitis” and asthma were part of the same disorder with “wheezy bronchitis” being at the milder end of the spectrum of severity. In the context of current practice, these children would now be regarded as those with intermittent asthma or viral-associated wheeze.

As part of the original study, 401 children were randomly selected from 30,000 Melbourne primary school children and were classified as follows: control group (C), 105 children who had never wheezed; mild wheezy bronchitis group (MWB), 74 children with fewer than 5 episodes of wheezing associated with bronchitis or respiratory tract infection; wheezy bronchitis group (WB), 104 children with 5 or more episodes of wheezing associated with bronchitis or respiratory tract infection; asthma group (A), 113 children with wheezing not associated with respiratory tract infection. When the children were reviewed at age 10 years, it was realized that there were very few with severe asthma, and a further sampling was performed from the same birth cohort to establish a severe asthma group (SA), 83 children with onset of symptoms before 3 years of age, persistent symptoms at 10 years of age, and a barrel-chest deformity or a reduction of FEV_0.5_/FVC ratio to 50% or less.

These subjects have been reviewed at ages ten, fourteen, twenty-one, twenty-eight, thirty-five, and forty-two years and most recently at 50 years of age making this study the longest, most comprehensive followup study of childhood asthma into adult life [6–11].

### 2.1. Outcomes of the Study

The overall participation rate ranged from 76% to 90% with good representation of the original cohort across the four decades. At the 50-year followup, 64% of the MWB/WB (intermittent asthma) groups, 47% of the A group and 15% of the SA group had achieved asthma remission. In the wheezy bronchitis groups, a majority of the remissions had occurred prior to age ten (46%). In the asthma group, remission occurred most commonly between the ages of 14 to 21 years. The remission rate for the severe asthma group remained low throughout the study periods. The main childhood predictors of asthma persistence at age 50 were the severe childhood asthma group, female sex, and childhood hay fever [11].

The reduction in lung function that occurred in the asthma and severe asthma group was established in early childhood with a similar trend throughout adult life. In those with wheezy bronchitis in childhood, lung function was preserved and is not different from controls. Importantly, there did not appear to be an increased rate of decline in lung function in the asthma and severe asthma groups when compared to the control and wheezy bronchitis groups despite the persistence of symptoms. This decline in lung function was not affected when variables such as gender and smoking behavior at the age of 21 or 50 years were taken into account.

The other major finding at the age of 50 years was that children with severe asthma were at significantly increased risk of developing adult chronic obstructive pulmonary disease but this was not the case for those with mild childhood asthma [12].

### 2.2. Strengths of the Study

The strength of this study has been the high level of participation which has been maintained throughout the last 4 decades. The comprehensive nature of testing involving consistent questionnaires, repeated lung function testing, and skin prick tests at each followup enabled accurate comparisons and detailed trends across the study periods.

### 2.3. Limitations of the Study

A major limitation of the study is the lack of information regarding pregnancy (e.g., smoking), birth (e.g., prematurity, birthweight), and early life events (e.g., infections, eczema). The other major limitation is the fact that children in the original cohort were managed in an era when appropriate medications such as inhaled corticosteroids were not available. Therefore it would not be possible to generalize the findings to current children with asthma. Data which is collected by questionnaire is clearly subject to memory recall error which may have incorrectly defined patient wheeze phenotype and smoking behaviour. Lastly, there were inconsistencies noted in the smoking data which meant that the true impact of smoking quantity and behaviour on lung function decline had to be interpreted with caution.

## 3. The Dunedin Multidisciplinary Health and Development Study (New Zealand Cohort)

This study was initiated as a New Zealand birth cohort study which has followed up children till the age of 38 years [2, 13, 14]. The birth cohort represented children born in Dunedin, New Zealand, in 1972-73. In the original study, 1139 children were born in that period of which 1037 (91%) participated in the first assessment at 3 years of age. The children were seen every 2 years from 3 and 15 years of age, and then at 18, 21, 26, 32 and most recently at 38 years. Respiratory questionnaires and lung function measurements were only initiated from the age of 9 years. In subsequent reviews, children underwent questionnaires, skin prick testing, and IgE measurement as tests for atopy, lung function testing, and methacholine bronchial challenge testing. Longitudinal lung function data of this study to the age of 26 years were published based on the original classification on childhood wheeze phenotype. Following the age of 26 years, lung function data to the most recent followup at 38 years have been published following a different classification and specifically addressing the influences of smoking behavior.

In this study, wheeze phenotype was defined as follows: persistent wheezing, wheezing reported at every assessment after it is first mentioned; remission, absence of wheezing after wheezing had been reported at two or more successive prior assessments; relapse, wheezing reported at two or more successive assessments and then absent at one or more successive assessments and then reported at all subsequent assessments; intermittent wheezing, presence of symptoms at some assessments but not the others; transient wheezing, wheezing reported at one assessment only.

### 3.1. Outcomes of the Study

In the initial assessment at 9 years of age, 815 study members (78.6% of the cohort of 1037) completed respiratory questionnaires. By the age of 26 years, 613 (59.1%) of the study members had provided respiratory data at every assessment. 27.4% of the study members had never reported wheezing at any time periods and 72.6% of the study members had reported wheezing during at least one assessment. 26.9% of the study members were currently wheezing at age 26 years with a majority either in remission or reporting intermittent wheezing. 12.4% had had a remission followed by a relapse by the age of 26 years. In 14.5%, wheezing had persisted from onset.

The risk factors that promote persistence of wheeze included being female, having a higher prevalence of sensitivity to House Dust Mite (HDM) and cat allergen, smoking at 21 years, and airway hyperresponsiveness. Those with asthma persistence and relapses had lower lung function measurements (expressed as the ratio of FEV_1_/FVC) at each assessment during childhood, adolescence, and adulthood compared to those who have never wheezed. There was also no statistical difference in the slopes of change in the FEV_1_/FVC ratio over time in any outcome category.

At the most recent followup at 38 years, 840 (81%) subjects were recruited and the cohort were reclassified to the following groups with childhood-onset persistent asthma, late-onset asthma, asthma in remission, and being nonasthmatic. Cumulative smoking history was associated with lower FEV_1_/FVC ratios among subjects without asthma at age 38 years and those with late-onset or remittent asthma but smoking was not associated with lower FEV_1_/FVC ratios among those with childhood-onset persistent asthma.

### 3.2. Strengths of the Study

This was a major unselected, population birth cohort study conducted and the sample at 38 years was representative of the original birth cohort. This was supported by the findings that there were no significant differences in sex ratio, family history of asthma and hay fever, symptoms, proportion with atopy, and lung function measurements between the participants and the original cohort of 1037. Identical challenge testing with methacholine was used consistently at each review to the age of 26 years, as a measure of bronchial hyperresponsiveness which enabled consistent comparison between groups at different time intervals. The findings of increased bronchial hyperresponsiveness in the persistent and relapsing groups suggest that active inflammation may be potentially occurring in the airways and that this was a risk factor for asthma persistence beyond childhood years. This study highlighted that children with more severe wheeze phenotypes (persistent wheezing) had reduced lung function when first measured at age 9 years, with no apparent increase in rate of decline when compared to all the other groups. At age 38 years, there was no evidence that cumulative smoking had a synergistic effect in the lung function decline in those with childhood persistent asthma.

### 3.3. Limitations of the Study

The histories of wheezing in early childhood were obtained from the parent when the children were already nine years of age. This raises the possibility of recall error such as underestimated reports of early wheezing. There was no description of pregnancy and early birth events in the studies either. Clearly, by the age of nine years, some children with early wheeze may have already resolved by the time when the respiratory questionnaires were conducted. The definition of remission is also subject to error as some subjects may be wheeze-free due to medication use which was not elaborated in the definition. By the age of 26, complete analysis was carried out on 59.1% of the subjects who had measurements at each study time period which may potentially lead to selection biases, although the participation rate of 81% at age 38 years is a marked improvement.

## 4. Tucson Children's Respiratory Study

This study recruited 1246 newborns born between May 1980 and October 1985 and has followed up the children into young adulthood. At the time of enrolment, the parents completed a questionnaire about their history of respiratory illness, smoking habits, and education. Parents completed a questionnaire during their child's second and sixth years of life. During the first year of life, 169 infants underwent pulmonary function testing involving partial expiratory flow-volume curves obtained by the chest compression technique. This technique measured the *V*_max_ FRC which was believed to reflect the size of the intrapulmonary airways [15]. At the time of the six-year survey, partial expiratory flow-volume curves were obtained with manouvers to measure voluntary maximal expiratory flow.

Skin prick testing was performed concomitantly with lung function testing at the time of the six-year survey with extracts of seven common aeroallergens. Total serum IgE levels were measured with the paper radioimmunosorbent test in samples obtained from cord blood, from blood obtained at a median age of 9.3 months, and from blood obtained at the time of the 6 year survey.

Wheeze phenotype at the assessment at 6 years of age was defined as follows: children who never had wheezing, no recorded lower respiratory tract illness with wheezing during the first three years of life and no wheezing at six years of age; transient early wheezing, children with at least one lower respiratory tract illness with wheezing during the first three years of life but who had no wheezing at six years of age; transient early wheezing was a term which was uniquely coined to reflect children with wheezing in early years of life which had ceased by 6 years of life; wheezing of late onset, those who had no wheezing during the first three years of age but who had wheezing at six years of age; persistent wheezing, those who had at least one lower respiratory tract illness with wheezing in the first three years of life and had wheezing at six years of age.

### 4.1. Outcomes of the Study

In the 6th year analysis [16], 51.5% had never wheezed, 19.9% had transient early wheezing, 15.0% had wheezing of late onset, and 13.7% had persistent wheezing. Risk factors that were associated with persistent wheezing included maternal asthma, maternal smoking, rhinitis apart from colds, eczema during the first year of life, male sex, and Hispanic ethnic background. Children with transient early wheezing reported higher rates of maternal smoking during pregnancy. Overall, wheezing in the first three years of life ran a rather benign course. Although one third of all children three years or younger had lower respiratory tract illnesses with wheezing, almost 60% of these children had stopped wheezing by the age of 6 years. Atopy was significantly more prevalent in both groups of children with wheezing at age of 6 than in the group that had never wheezed. Children with persistent wheezing had significantly higher IgE levels at nine months of age than those who had never wheezed.

This birth cohort has been recently followed up to early adult life at the age of 22 years. Children who had infant lung function in the lowest quartile also had lower prebronchodilator values for FEV_1_/FVC ratio, FEF_25–75_ and FEV_1_ up to age 22 years. Results in this study suggest that poor airway function shortly after birth should be recognized as a risk factor for airflow obstruction in young adults and these subjects may reach the threshold of FEV_1_ and FEV_1_/FVC ratio that define chronic obstructive pulmonary disease (COPD) at an earlier age [17].

### 4.2. Strengths of the Study

This is the longest prospective birth cohort study which has followed up children from birth to young adulthood. The Tucson cohort has also been instrumental in defining three key phenotypes of asthma: transient wheezing of infancy, nonatopic wheezing, and IgE mediated wheezing. The uniqueness of lung function measurements from the 1st year of life to young adulthood has demonstrated that lung function was partly determined in early life, therefore highlighting the possibility of in-utero effects and the risks associated with maternal smoking in pregnancy. The most recent followup in young adulthood also reports the possibility that adult chronic obstructive pulmonary disease (COPD) may have its origins in early life.

### 4.3. Limitations of the Study

The participation rate of the subjects has decreased throughout the study periods which raise the possibility of selection bias. Reports on symptoms are based on questionnaires which are predisposed to memory recall error. There are inherent limitations in the measurement of *V*_max_ FRC such as instability on FRC in infants with disease or sleep and that infants rarely exhale to residual volume using this technique. This method was applied in the early phase of the study and would now be regarded as an inferior technique to the raised volume rapid thoracoabdominal compression technique [18]. Nevertheless, the results that have been generated from this birth cohort have provided significant information regarding the lung function during the early years of life not previously attained by previous studies.

## 5. Tasmania Asthma Study

In 1968, the parents of all 8683 children born in 1961 and attending school in Tasmania were asked to complete questionnaires on their children's history of respiratory symptoms and asthma. The survey aimed to study the natural course of asthma and returns from 99% of children were received [19]. In the original study, children for whom the answer to “has he or she at any time in his or her life suffered from attacks of asthma or wheezy breathing?” was “yes” were considered to have had childhood asthma. Questions were also asked about hay fever, eczema, and parental smoking. Lung function measurements included FEV_1_/FVC and FEF_25–75_. During 1991–1993, 2000 subjects from the 1961 birth cohort were selected at random for a followup study. This included 1000 from the 1349 subjects who had childhood asthma and 1000 from 6993 who did not have childhood asthma [4]. In the followup study, “current asthma” was defined as the occurrence of an attack of asthma within the previous 12 months. “Current atopic asthma” was current asthma with current hay fever or eczema and “frequent asthma” was the occurrence of more than 10 attacks in the previous 12 months.

From 2002 to 2005, 7312 (85.2%) of the original participants were sent a detailed respiratory questionnaire which was completed by 5729 (78.4%) of subjects. A sample of respondents enriched for asthma and chronic bronchitis participated in lung function testing (including pre- and postbronchodilator spirometry) and skin prick testing as a measurement for atopy from 2006 to 2008 [20].

### 5.1. Outcomes of the Study

In the 1991–93 followup study, questionnaires were returned by 1494 subjects representing 74.7% of the total sample (75.1% of those with childhood asthma and 75.3% of those without). Interestingly, almost half of the subjects gave responses that contradicted those of their parents in 1968 with regards to onset of asthma by age of 7. Of the subjects with asthma or wheezy bronchitis by the age 7, as reported by their parents 25.6% reported current asthma as an adult. 75% of the children with asthma reported by their parents in 1968 were free of symptoms as adults, consistent with asthma remission. The risk factors measured at the age of 7 that independently predicted current asthma as an adult were being female, having a history of eczema, having a low mid forced expiratory flow, having a mother or father with a history of asthma, having childhood asthma, and having the first attack by the age of 2 or having had more than 10 attacks [4]. Childhood allergic rhinitis defined by an affirmative answer to “does he/she get attacks of ‘hay fever' (i.e., sneezing, running or blocked nose, sometimes with itchy eyes or nose)?” before the age of 7 was associated with a 3-fold increased risk of childhood asthma persisting by middle age and a significant increased risk of incident asthma in preadolescence, adolescence, or adult life [21].

In the 2002–05 followup study, the authors report that childhood infectious diseases (such as rubella, chicken pox, and pertussis) appear to protect against asthma persisting in later life, although pertussis and measles were associated with new-onset asthma after childhood [22]. Asthma remission was also more likely to occur in those with childhood-onset asthma and less likely in those who are females and those with impaired lung function at the age of 7 years, allergic rhinitis, and eczema, which were similar to the previous survey. Asthma remission was interestingly not affected by adult smoking, passive smoking, or childhood exposure to parental smoking [23]. In the most recent followup of 1389 subjects from 2006 to 08, fixed airway obstruction was demonstrated in 6.0% of the cohort. The authors report a three-way interaction between the effects of clinical asthma, active smoking, and atopy on the higher risk of fixed airways obstruction [20].

### 5.2. Strengths of the Study

There was a high participation rate (75%) at the followup study, with large numbers of subjects, which was representative of the birth cohort and limits response bias. This study has also highlighted the key risk factors of parental asthma, frequent childhood asthma episodes, and rhinitis as determinants of asthma persistence from childhood to adult life. This study has also provided helpful information to suggest that rhinitis in childhood is a risk factor to the development of new-onset adult asthma [21].

### 5.3. Limitations of the Study

There were significant differences in the parents' report of symptoms when the children were 7 years of age with the subjects' report in the followup study indicating the error of recall and unreliable nature of retrospective self-assessment of childhood asthma in adults [24]. The definition of asthma involving the use of the word “attack” may have underestimated subjects who only had periodic episodes of wheeze rather than true “attacks” of asthma. There was no objective testing performed to assess atopy status in the studies which limits its measurement as a valid test for analysis.

## 6. British National Child Development Study

This birth cohort study of Perinatal Mortality started in 1958 with a focus on 18,559 births in a single week [25]. The original study aimed to identify social and obstetric factors linked to stillbirth and neonatal death. The National Children's Bureau was commissioned by the Central Advisory Council for Education to retrace the cohort at age 7 and prospectively monitor their educational, physical, and social development at ages 7, 11, 16, 23, 33, and most recently 45 years. At age 7, 14,571 (79%) contributed information on asthma and bronchitis with wheezing. Asthma was defined by a positive answer to “has your child ever had attacks of asthma?” Wheezy bronchitis was defined by a positive answer to “has your child ever had attacks of bronchitis with wheezing?” At age 33, wheezing illness was defined by a positive answer to “some people feel that their chest is sometimes wheezy or whistling; have you had wheezing or whistling in your chest at any time in the past? If yes, have you had any wheezing or whistling in your chest at any time in the past 12 months?”

### 6.1. Outcomes of the Study

5,801 (31%) subjects contributed data at ages 7, 11, 16, 23, and 33 years. Persistence of wheezing at each followup was assessed by responses indicating one or more attacks of asthma or wheezy bronchitis in the previous year. The cumulative incidence of wheezing illness was 17% by age 7, 24% by age 16, and 43% by age 33. Incidence during childhood was strongly associated with pneumonia, hay fever, and eczema. Relapse at 33 after prolonged remission of childhood wheezing was more common among current smokers and atopic subjects. A quarter of children with a history of asthma or wheezy bronchitis by age 7 reported wheeze in the previous year at the age of 33.

From 1992 to 1993, lung function with postbronchodilator responses was performed in 1060 subjects with a history of asthma, wheezy bronchitis, or wheezing and 275 controls. The adults were aged between 34 and 35 years. Among 551 cases reporting no wheeze in the year before examination, ventilatory function did not differ significantly from the control, except for FEV_1_ in 192 subjects with wheezing before the age of 7. Among 509 cases reporting wheeze in the past year, FEV_1_ and FEV_1_/FVC ratio were reduced to a greater extent in those with an earlier age of onset of wheeze highlighting the importance of childhood asthma as a determinant of adult lung function [26]. Approximately 91% of the 1392 cohort members who undertook spirometry at age 35 years were reexamined at age 45 and 83% were included in the analysis [3]. Subjects who suffered wheeze before the age of 7 years showed the steepest rate of decline of FEV_1_ (36.3 mL/yr) compared with other transient, persistent, and never wheezers (33.5 mL/yr). Smokers at age of 42 years suffered a higher rate of decline in FEV_1_ between the ages of 35 and 45 than former or never smokers (*p* = 0.008).

### 6.2. Strengths of the Study

The main strengths of the study include the largest prospective followup study of children from a birth cohort and a thorough assessment of new incident cases of asthma in adult life describing smoking and atopy as major risk factors. The British cohort also described smoking to be associated with an increase in the rate of decline in lung function, which was one of the few longitudinal studies to describe this additional decline in lung function.

### 6.3. Limitations of the Study

The response rate at the 33-year-old study had dropped to 31% reflecting significant loss over time primarily by relocation. The definitions of asthma and wheezy bronchitis were based on the term “attacks” which may have underestimated subjects with “wheeze” alone. Lung function was firstly measured at age 33 years with no prospective measurements made in childhood. There were also no objective markers of atopy throughout the study limiting its assessment.

## 7. Lessons to Be Learnt from the Cohort Studies

The clinical and lung function outcomes of adults with asthma appear to be partly established in childhood years. Children with mild asthma are more likely to achieve remission and those in the severe phenotype are more likely to have ongoing symptoms into adult life. Some of the main risk factors that predispose children to persistence of asthma symptoms into adult life include female sex, parental history of asthma, and atopy in particular allergic rhinitis.

There have been studies that describe increasing prevalence of asthma in females from adolescence into adulthood [27–29]. Female sex has been found to be a risk factor determining asthma persistence from childhood into adult life in the Melbourne, New Zealand, and Tasmanian cohorts [2, 4, 28, 29]. Various factors have been suggested to explain the gender differences in asthma prevalence and incidence such as hormonal modulation or gender difference in hyperreactivity of the airways but this relationship remains ill-defined and unclear [30, 31]. A recent review on the effects of gender and asthma suggests that androgens were likely to have an immunosuppressive effect whilst estrogens were proinflammatory and might increase the atopic susceptibility [32]. This view is likely to be overly simplistic and more studies are required to clarify the regulatory effects of sex hormones in the persistence of asthma in females.

It is interesting to speculate on the “one airway, one disease” hypothesis as a basis to explain the association between allergic rhinitis and asthma [33]. Other mechanisms that have been suggested include the possible inhalation of inflammatory cells from the upper airway into the lower airways resulting in clinical asthma [34]. Inflammatory changes seen in the nasal and bronchial mucosa have been found to be similar whereby eosinophils, mast cells, T cells, and macrophages are found. Likewise, inflammatory cytokines such as IL-4, IL-5, and IL-13 which promote eosinophilic inflammation are also noted in both mucosae [35]. Mechanisms to explain the temporal relationship between the two diseases require further clarification to determine if allergic rhinitis predisposes and precedes asthma, triggers asthma, or worsens asthma.

Lung function data from the Melbourne, Dunedin, and Tucson cohorts indicates that children with more severe asthma achieve suboptimal lung growth. The comprehensive lung function measurements in the Melbourne and Dunedin cohorts demonstrate a parallel decline in lung function across the severe asthma groups which was not different when compared to the control and mild asthma groups. This finding is contrary to publications from adult based asthma cohorts. The adult Busselton study was a multiple cross-sectional study where asthma was acquired at any time during the study period. The reduction in lung function that occurs with the onset of asthma during a longitudinal study would influence the rate of decline over the length of the study period [36]. The synergistic impact of smoking and asthma on lung function has been demonstrated in the adult studies as well. This has not been described in longitudinal childhood asthma studies. The combination of asthma and smoking > 15 cigarettes per day (*n* = 101) had a synergistic effect on the decline in lung function and resulted in a 17.8% decline in FEV_1_ over 10 years in the CARDIA cohort [37]. In the Melbourne cohort smoking behaviour did not accelerate the decline in lung function although the reported smoking behavior suggests that this data should be interpreted with caution.

The results of the Melbourne cohort suggests that children with severe asthma or those with deficits in lung function from childhood are clearly at increased risk of fulfilling the diagnosis of adult chronic obstructive pulmonary disease [12]. It is important to acknowledge that these children with severe asthma were managed in an era when effective treatment such as inhaled corticosteroids was not available. Clearly it would be of interest to follow up current birth cohorts into adult life to examine the likelihood of this association as children with severe asthma are now managed accordingly with inhaled corticosteroids.

Given the findings of the childhood asthma cohorts, it would support the focus of studies in the preschool years which appear to be the period when changes of airway remodeling might be occurring. Future research needs to continue into understanding the mechanisms that precede airway remodeling. Ideally, airway epithelial biopsies would provide the most information but given the invasive nature of such a procedure, further noninvasive tools need to be developed. High resolution CT (HRCT) scanning in adults has shown good correlation between spirometric indices of airflow obstruction, reticular basement membrane thickness, and HRCT measurements of airway wall thickness [38] but this has not been established in children [39].

Lastly, other noninvasive techniques to measure airway inflammation and biomarkers should be explored in preschool children. Exhaled breath condensate (EBC) is a simple tool which may be of use in preschool children abut at this stage; no useful biomarkers for remodeling have been identified. Salivary proteins may also be a useful tool in determining candidate proteins that identify children who may have an atopic tendency and predisposition to asthma.

## Figures and Tables

**Table 1:** Characteristics of longitudinal cohorts.

	Age of recruitment (yrs)	Most recent followup age (yrs)	Number of original subjects	Recent followup (participation rate)	Atopy measurements	Lung function measurements (years)
Melbourne cohort	7	50	484	346 (76%)	Eczema, allergic rhinitis, skin prick tests	7, 10, 14, 21, 28, 35, 42, 50 years
Dunedin cohort	9	38	1139	840 (81%)	Eczema, allergic rhinitis, skin prick tests	9, 11, 13, 15, 18, 21, 26, 38
Tucson cohort	Birth	22	1246	123/169 (73%) who had infantile lung function	Eczema, allergic rhinitis, skin prick tests, serum IgE	1 (*V*_max_ FRC), 11, 16, 22
Tasmanian cohort	7	45–47	8683	1389 (selected cohort)	Eczema, allergic rhinitis, skin prick test	13, 30, 45–47
British cohort	7	45	14571	1266 (selected cohort)	Not collected	34-35, 45
